# A Change in Global Sagittal Alignment after Transforaminal Epidural Steroid Injections in Lumbar Spinal Stenosis

**DOI:** 10.3390/jcm12144727

**Published:** 2023-07-17

**Authors:** Park Manakul, Koopong Siribumrungwong, Naphakkhanith Dhanachanvisith

**Affiliations:** 1 Department of Orthopedics, Faculty of Medicine, Thammasat University, Pathum Thani 12120, Thailand; poneoz@hotmail.com (P.M.); best_072@hotmail.com (N.D.); 2 Chulabhorn International College of Medicine, Thammasat University, Pathum Thani 12120, Thailand

**Keywords:** global sagittal balance, transforaminal epidural steroid injections, lumbar spinal stenosis

## Abstract

**Patients’ functional adaptation to pain can affect global sagittal alignment.** This study evaluated the short-term spinal sagittal alignment change after transforaminal epidural steroid injection (TFESI) in lumbar spinal stenosis patients. Patients with lumbar spinal stenosis who underwent TFESI were retrospectively examined. Clinical outcomes were assessed using the Visual Analog Scale (VAS) and Oswestry Disability Index (ODI). Before and two weeks after the intervention, whole-spine lateral standing view radiographs were taken. Radiographic parameters including the Sagittal Vertical Axis (SVA), C2C7 Cobb, Thoracic Kyphosis (TK), Lumbar Lordosis (LL), Pelvic Incidence (PI), Pelvic Tilt (PT), Sacral Slope (SS), and Lumbopelvic Mismatch (PI-LL) were measured. Ninety-nine patients (mean age 64.3 ± 9.2 years) were included in this study. Both VAS and ODI outcomes were statistically improved after two weeks of intervention. Radiographic parameters showed that SVA, PT, and PI-LL mismatch were significantly decreased, while C2C7 Cobb, TK, SS, and LL were significantly increased after the intervention. SVA was improved by 29.81% (52.76 ± 52.22 mm to 37.03 ± 41.07 mm, *p* < 0.001). PT also decreased significantly from 28.71° ± 10.22° to 23.84° ± 9.96° (*p* < 0.001). Transforaminal epidural steroid injection (TFESI) significantly improves VAS, ODI, and global sagittal parameters in lumbar spinal stenosis patients.

## 1. Introduction

Global sagittal balance is important for patient well-being. Previous studies have reported that spinal imbalance causes more muscle energy expenditure [1] to maintain body balance and movement, resulting in pain, fatigue, and disability [2,3]. For this reason, accurate planning for global sagittal alignment correction is essential for spinal surgeons to achieve the best outcomes and improve patients’ quality of life [4].

Other than structural spinal deformity, which causes global spinal imbalance, a patient’s functional adaptation can also affect global sagittal alignment [5]. Lumbar canal stenosis may compensate for the pain they feel by leaning forward or adopting a flexion posture. This has been proven to alleviate pain due to an increase in the spinal canal diameter [6,7]. From previous studies, spinal surgical procedures for treating spinal canal stenosis, such as decompression alone [8], discectomy [9,10], or short-segment fusion [11], result in an improvement in sagittal spinal imbalance. However, to our knowledge, no study is yet to report the effect of transforaminal epidural steroid injection (TFESI) in sagittal spinal alignment.

TFESI is recognized as a conservative treatment in lumbar spinal stenosis to alleviate symptoms [12,13], and as a diagnostic procedure [14] to identify pathologic levels in complex cases. TFESI is commonly used as an initial procedure before considering surgical management, and this study aims to evaluate the short-term spinal sagittal alignment change after TFESI. 

## 2. Materials and Methods

All patients provided written informed consent. We retrospectively reviewed cases from electronic medical records and radiographs of lumbar spinal stenosis patients aged between 18 and 85 years old at Thammasat University Hospital, who underwent transforaminal epidural steroids injections from January 2017 to January 2020. The inclusion criteria were patients who have a history of clinical radiculopathy from spinal stenosis and lumbar disc herniation with complete pre-intervention and post-intervention data records. The exclusion criteria were spinal infection, clinical progressive neurologic deficit, symptoms of cauda equina or conus medullaris syndrome, a history of spinal surgery, ankylosing spondylitis, active hip disease, and a history of other conditions that can mimic spine pathology (such as urologic, gynecologic, or great vessel disease).

Demographic data were collected from the electronic medical records, including age, sex, and the vertebra level injected. Clinical outcomes were recorded pre-intervention and two weeks after intervention. The Visual Analog Scale (VAS) and Oswestry Disability Index (ODI) (Thai version) [15] were collected.

### 2.1. Radiological Measurement

Whole-spine anteroposterior (AP) and lateral standing radiographs were taken before intervention (pre-intervention) and two weeks after intervention (post-intervention) by using 36-inch full-length films. To standardize the imaging process, all patients were instructed to stand in a comfortable position with full hip and knee extension, and with the elbow flexed at 45° [16].

**Figure 1:**
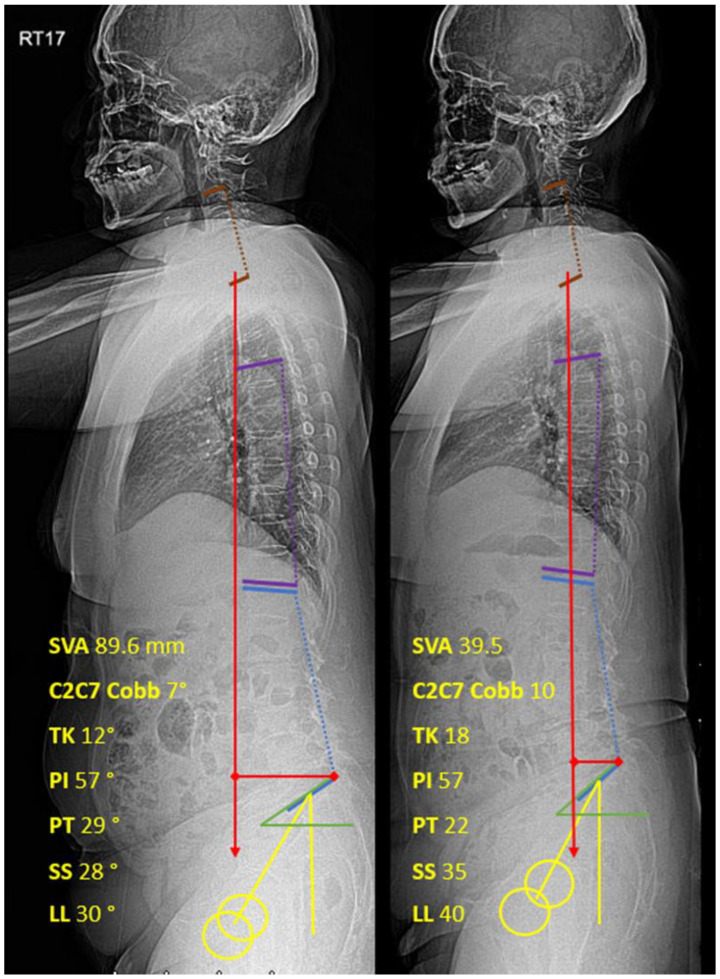
Whole-spine lateral standing films with measured sagittal parameters ((**left**): pre-intervention; (**right**): post-intervention). Red: Sagittal vertical axis, Purple: Thoracic kyphosis, Blue: Lumbar lordosis, Yellow: Pelvic tilt, Green: Sacral slope.

Two spine surgeons independently performed digital radiograph interpretations. The patients’ data and identifications were blinded to the evaluators. The sagittal parameters were measured by PACS (SYNAPSE, Fujifilm’s) measurement tools on the 27-inch monitor. The Sagittal Vertical Axis (SVA), Thoracic Kyphosis (TK), Lumbar Lordosis (LL), Pelvic Incidence (PI), Pelvic Tilt (PT), Sacral Slope (SS), and PI-LL (PI minus LL) were measured according to the Scoliosis Research Society-Schwab classification [1]. C2C7 Cobb was measured from the angle between C2 and the C7 lower endplate [17] (Figure 1).

### 2.2. Intervention

**Figure 2:**
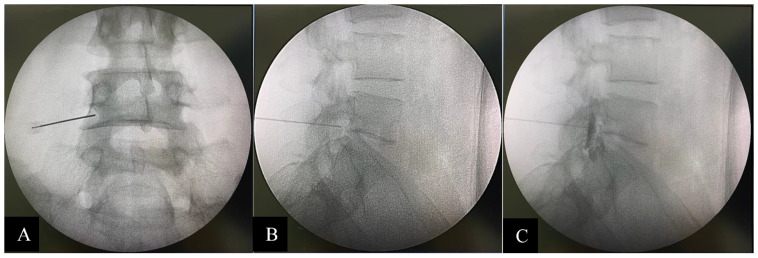
(**A**) AP fluoroscopic view. The end of the needle is located in a safe triangle. (**B**) Lateral fluoroscopic view. The end of the needle is located in the middle of the neural foramen. (**C**) Lateral fluoroscopic view. After the L4 nerve root, contrast injection was outlined.

TFESI was performed with triamcinolone acetonide 40 mg/1 cc (40 mg for one-level injection and 80 mg for two- to four-level injection) and normal saline mixed up to 2 cc for each injection point. In addition, 0.5–1 cc of Iohexol (Omipaque 300 Contrast) was administered as a contrast media to confirm the position prior to steroid injection under biplanar (AP and lateral) fluoroscopic guidance (C-arm Fluoroscope, Philips BV Pulsera, Amsterdam, The Netherlands). Quincke spinal needles, size 23, gauge 9 cm, were used, and they were angled towards the safe triangle [18,19] in the AP view and towards the middle of the neural foramen in the lateral view (Figure 2).

### 2.3. Statistical Analysis

Percentages were used for categorical data. The mean and standard deviation were used for continuous data after the normality assumption was validated, and a paired *t*-test was used in the analysis. Characteristics of the data between groups were analyzed by analysis of variance (ANOVA). The correlation of the data was measured using Pearson correlation coefficients. An r value of more than 0.3 or less than −0.3 confirmed a statistical correlation [20], and a *p* value of less than 0.05 indicated statistical significance. Interobserver reliability testing was performed using the Intraclass Correlation Coefficient (ICC). All statistical calculations were performed on IBM SPSS Statistic version 25.0 (IBM Corporation, Armonk, NY, USA).

## 3. Results

We retrospectively reviewed 120 individual cases with 99 patients. A total of 21 males (21.2%) and 78 females (78.8%) underwent TFESI and were included in the study. The average age was 64.3 ± 9.2 years. The average vertebrae levels injected were 1.8 ± 0.8 levels.

### 3.1. Pre-Intervention and Post-Intervention Outcomes

Both VAS and ODI outcomes were statistically improved after the intervention: VAS decreased from 8.31 ± 1.11 to 3.38 ± 1.47 and ODI decreased from 31.06 ± 3.48 to 18.03 ± 4.17. All sagittal parameters were statistically significantly improved. SVA, PT, and lumbopelvic mismatch were significantly decreased, while C2C7 Cobb, TK, SS, and LL were significantly increased after the intervention. Post-intervention SVA showed an improvement of 30.12% (53.26 ± 51.67 mm to 37.22 ± 40.71 mm). Pelvic Incidence (PI) was not statistically affected by TFESI. All data are shown in Table 1.

### 3.2. Difference between the Number of Injection Levels and Sagittal Parameters

The collected data were divided according to the total number of vertebrae levels injected: 1 level, 2 levels, 3 levels, and 4 levels. The differences in outcomes and sagittal parameters in each group were assessed. The variations of VAS score and ODI among the different numbers of injection levels were not statistically significant, both before and after the intervention. The C2C7 Cobb and TK were found to not be statistically significant between injection levels. The sagittal parameters, including SVA, PT, and PI-LL mismatch, were statistically significantly higher in multiple-level injection groups compared to single-level injection groups, both before and after the intervention. In contrast, SS and LL were statistically significantly lower in multiple-level injection groups compared to single-level injection groups, both before and after the intervention. All data are shown in Table 2.

### 3.3. Correlation between Sagittal Parameters

**Figure 3:**
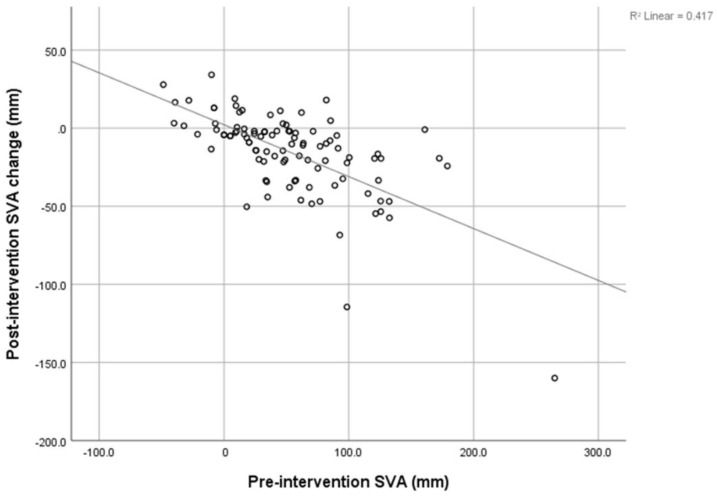
Correlation between pre-intervention SVA and post-intervention SVA change.

**Figure 4:**
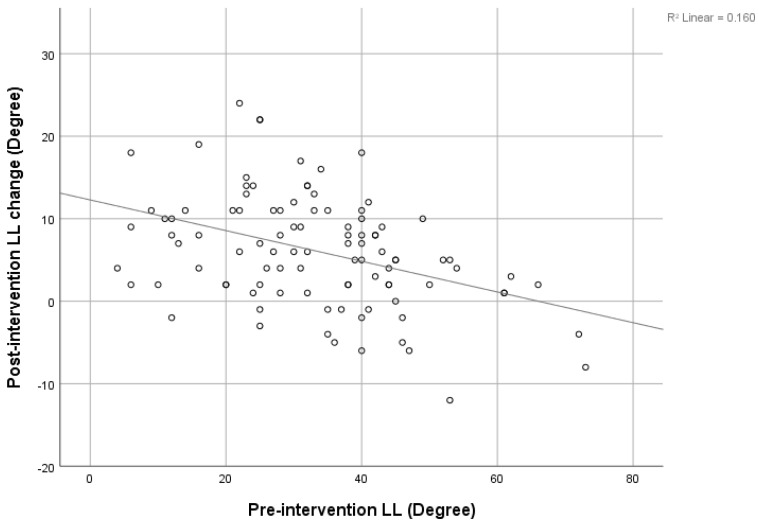
Correlation between pre-intervention LL and post-intervention LL change.

**Figure 5:**
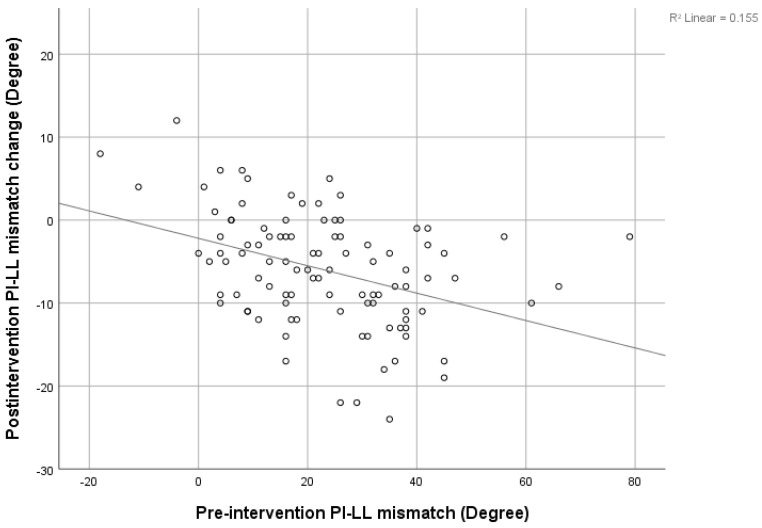
Correlation between pre-intervention PI-LL mismatch and post-intervention PI-LL mismatch change.

There was a strong negative correlation between the pre-intervention SVA and post-intervention SVA decrement (r = −0.646), shown in Figure 3. The pre-intervention LL showed a negative correlation with the post-intervention LL increment, shown in Figure 4 (r = −0.4). The pre-intervention PI-LL mismatch exhibited a negative correlation with the post-intervention PI-LL decrement (r = −0.394), shown in Figure 5. The significant correlation between pre-intervention parameters and post-intervention changes is shown in Table 3.

### 3.4. Interobserver Reliability 

Interobserver reliability was calculated using the Intraclass Correlation Coefficient. The results were greater than 0.9 in all sagittal parameters. Thus, it was considered that the measurements were valid and achieved excellent reliability.

### 3.5. Case Example

**Figure 6:**
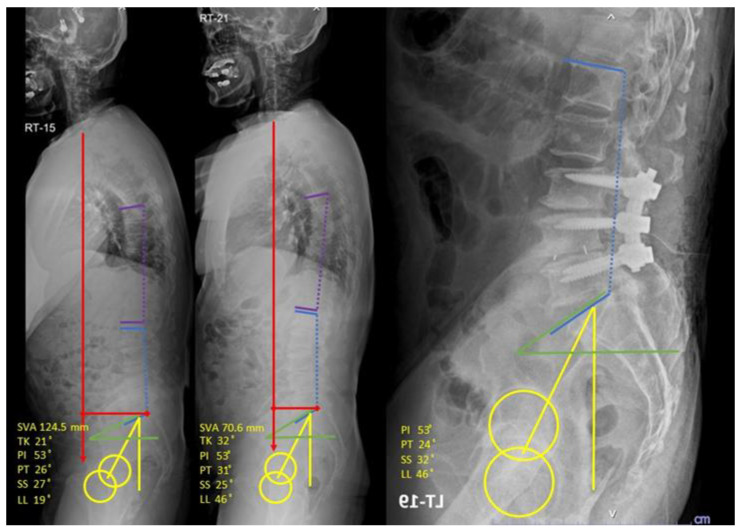
Whole-spine lateral standing films and post-operative films with measured sagittal parameters ((**left**): pre-intervention; (**middle**): post-intervention; (**right**): post-operative). Red: Sagittal vertical axis, Purple: Thoracic kyphosis, Blue: Lumbar lordosis, Yellow: Pelvic tilt, Green: Sacral slope.

A 63-year-old Thai man with low back pain and positive balance had pre-intervention parameters: SVA 124.5 mm, TK 21°, PI 53°, PT 26°, SS 27°, and LL 19°; and post-intervention parameters: SVA 70.6 mm, TK 32°, PI 53°, PT 31°, SS 25°, and LL 46°. After post-intervention parameters were evaluated, the operative planning was changed from long-segment fusion for correct SVA and multiple osteotomies for correct LL to short-segment fusion and single-level osteotomies instead (Figure 6).

## 4. Discussion

In our study, we collected data from patients with lumbar spinal stenosis who received TFESI. The results showed that both the VAS and the ODI improved significantly after the intervention. Ghahreman A. [21], Kabatas S. [22], and McCormick Z. [23] studied the short-term effects of TFESI, which significantly improved VAS and ODI in a similar study. The study of Karppinen et al. [24] showed that at 2 weeks of follow-up, a significant improvement from baseline was observed in every outcome parameter (leg pain, back pain, ODI, degree of straight-leg-raising test). This could support the treatment of pain using TFESI for short-term efficacy in lumbar spinal stenosis.

Liang C. [10] conducted a study on lumbar disc herniation and found significant immediate improvement in all sagittal imbalance parameters on day one post-operation, and this improvement continued for three months before the parameters became close to their normal ranges. SVA improved from 11.6 ± 6.6 cm to 2.9 ± 6.1 cm, and three months post-operation, LL improved from 25.3° ± 14.0° to 42.4° ± 10.2°. Moreover, Fujii K. [8] retrospectively reviewed lumbar decompression without fusion in lumbar spinal stenosis and concluded that the SVA, TK, PT, LL, and PI-LL mismatch improved post-operatively. SVA was decreased from 49.1° ± 38.6° to 28.6° ± 30.7°, and LL was changed from 38° ± 13° to 44° ± 11°. A strong correlation was found between pre-op SVA/PI-LL and post-op SVA/PI-LL decrement post-operatively. Likewise, Salimi H et al. [25] also reported that minimally invasive lumbar decompression surgery could convert sagittal malalignment to normal alignment in 2 years and 5 years follow-up. The previous studies mentioned above indicated that spinal decompressive procedures without instrumentation have the ability to improve sagittal spinal parameters. Therefore, we believe that spinal interventions that reduce radicular pain, such as TFESI, can partially improve sagittal spinal parameters because when radicular pain was improved, compensating forward bending subsided. 

This study may be the first study to collect data on the non-operative management of lumbar spinal stenosis. The present procedure did not interfere with the anatomical structure, but decreased the inflammation process to the neural structure and improved radicular pain. We found a significant change in SVA, C2C7 Cobb, TK, PT, SS, and LL after patients underwent TFESI. The SVA was improved by about 30% (from 52.76 ± 52.22 mm to 37.03 ± 41.07 mm), and PT and LL were improved by about 17% and 18%, respectively. This is compared to 42%, 15%, and 16% improvements reported by Fujii K. [8]. Patients with multiple levels of stenosis tend to have significantly more severe positive SVA, higher PT, and lower LL and SS. Furthermore, we found a strong negative correlation between pre-intervention SVA and post-intervention SVA decrement. This indicates that the more positive imbalanced patients were, the greater the resulting improvement in the SVA. This correlation has also been found in recent studies [8,10,26]. Similarly, a strong negative correlation between pre-intervention PI-LL and post-intervention PI-LL was found. The greater the PI-LL mismatch, the larger the PI-LL improvement could be predicted to be. In contrast, the pre-intervention LL was negatively correlated with the post-intervention LL increment, and this means that in a small cohort of pre-intervention LL patients, there may be more improvement in the post-intervention LL.

From a review of previous literature, it can be observed that many spinal pathologies are caused by sagittal imbalance, reduced muscle strength [10,27,28], adjacent disc degeneration [29], disc herniation [9], and spinal stenosis. Several authors have proposed that spinal stenosis patients have limited lumbar lordosis (LL) [5] due to the decreased pressure of the epidural venous plexus when bending forward. Furthermore, compensatory lumbar flexion posture lowers epidural pressure, thus reducing pain and neurogenic claudication [9,27,30,31]. The anatomical study showed that flexion for the lumbar spine increased spinal canal diameters [6,7]. We hypothesized that in global sagittal imbalance patients, there might be two factors that are involved in the imbalance. The first one is a structural imbalance, and the second one is the “functional compensation” of patients to radicular pain. We believe that after undergoing TFESI and the pain becoming less severe, compensation of lumbar flexion may be diminished. In this study, after TSESI, we found that SVA, PT, and LL were significantly improved. Recently, there has been little focus on the functional compensation of sagittal alignment before spinal surgical correction. We believe that it is better to evaluate the spinal surgical balance when the clinical pain of patients is subsiding, rather than when the pain remains severe.

Our most recent concern with this main issue was that we were uncertain whether we had to correct the deformity if a global sagittal imbalance existed in the surgical treatment of degenerative lumbar spinal stenosis. This study found that a considerable number of patients’ global sagittal alignment significantly improved following TFESI. For this reason, in patients with degenerative lumbar spinal stenosis and global sagittal imbalance, reassessing global sagittal alignment after TFESI might show more accurate structural global sagittal imbalance. We advise obtaining whole-spine AP and lateral standing radiographs again after patients begin improving in terms of pain following TFESI.

This study has some limitations. First, this is a retrospective review of the database, so recall bias and selection bias may be present. Second, this radiographic study focuses on sagittal alignment, but the dynamic compensation of lower limbs, such as hip and knee flexion, is not investigated. It should be noted that we instructed all patients who received the whole-spine film to extend their hip and knee before imaging [32]. Third, due to the short-term effect of TFESI, the outcome and sagittal parameter data were collected only at a short-term follow-up. We suggest a long-term follow-up in future studies.

## 5. Conclusions

Transforaminal epidural steroid injection (TFESI) can improve SVA, C2C7 Cobb, TK, PT, SS, and LL parameters, as well as VAS and ODI, in a short-term follow-up study and also has benefits in that it is effective in correcting functional compensation to evaluate sagittal alignment correction before surgery to avoid postoperative overcorrection alignment. However, this could be the choice of treatment to improve quality of life factors in terms of pain and disability in sagittal malalignment patients who have contraindications or deny surgery.

## Figures and Tables

**Table 1:** Pre-Intervention and Post-Intervention Outcomes and Sagittal Parameters.

Parameter	Pre-Intervention	Post-Intervention	*p*-Value ^α^
**VAS**	8.31 ± 1.11	3.38 ± 1.47	<0.001
**ODI**	31.06 ± 3.48	18.03 ± 4.17	<0.001
**SVA, mm**	53.26 ± 51.67	37.22 ± 40.71	<0.001
**C2C7 Cobb**	12.15 ± 10.86	13.63 ± 10.26	0.004
**TK ^๐^**	19.22 ± 10.80	20.9 ± 10.46	0.001
**PI ^๐^**	56.25 ± 10.18	56.31 ± 10.13	0.296
**PT ^๐^**	28.65 ± 10.17	23.66 ± 9.96	<0.001
**SS ^๐^**	27.61 ± 9.00	32.65 ± 8.36	<0.001
**LL ^๐^**	33.35 ± 14.80	39.56 ± 13.34	<0.001
**PI—LL ^๐^**	22.89 ± 16.35	16.75 ± 14.82	<0.001

^α^ Calculated with paired *t*-test.

**Table 2:** Outcomes and Sagittal Parameters between Number of Injection Levels.

Parameter		Number of TFESI Injection Levels				
		1 (*n* = 40)	2 (*n* = 42)	3 (*n* = 14)	4 (*n* = 3)	*p* Value ^α^
**Pre-intervention**	VAS	8.30 ± 1.04	8.17 ± 1.15	8.71 ± 1.14	8.67 ± 1.53	0.42
	ODI	30.93 ± 2.76	30.74 ± 3.78	32.29 ± 3.79	31.67 ± 7.23	0.53
	SVA	25.59 ± 33.29	64.08 ± 45.29	72.44 ± 57.25	164.76 ± 98.35	<0.001
	C2C7 cobb	12.05 ± 8.45	11.12 ± 10.93	13.57 ± 6.76	19.00 ± 39.15	0.619
	TK	19.40 ± 8.90	16.60 ± 11.45	18.21 ± 12.25	10.00 ± 8.71	0.487
	PI	56.55 ± 8.59	53.81 ± 9.31	60.57 ± 12.15	67.33 ± 13.61	0.027
	PT	2.45 ± 9.65	28.19 ± 8.78	36.14 ± 9.21	44.61 ± 13.78	<0.001
	SS	31.13 ± 8.26	25.69 ± 9.18	24.43 ± 7.98	22.67 ± 0.57	0.011
	LL	39.5 ± 13.57	30.90 ± 12.76	27.0 ± 15.80	12.33 ± 1.52	<0.001
	PI-LL	17.05 ± 15.38	22.90 ± 12.46	33.57 ± 16.79	55.0 ± 14.93	<0.001
**Post-intervention**	VAS	3.17 ± 1.50	3.37 ± 1.48	3.69 ± 1.10	5.00 ± 1.73	0.17
	ODI	17.47 ± 3.70	17.69 ± 4.42	19.84 ± 3.93	22.33 ± 4.93	0.08
	SVA	19.34 ± 28.96	44.8 ± 39.30	53.22 ± 47.44	97.76 ± 64.28	0.0002
	C2C7 cobb	13.70 ± 8.30	13.27 ± 10.67	13.5 ± 8.46	17.83 ± 31.05	0.907
	TK	21.83 ± 8.60	21.83 ± 10.93	18.19 ± 13	6.83 ± 4.25	0.071
	PI	56.7 ± 8.78	54.13 ± 9.98	59.84 ± 12.5	67.16 ± 11.62	0.067
	PT	20.8 ± 8.73	23.40 ± 9.24	29.00 ± 10.57	41.00 ± 10.44	0.0007
	SS	35.88 ± 6.75	30.60 ± 8.84	30.84 ± 9.22	26.16 ± 1.60	0.011
	LL	43.78 ± 11.85	38.61 ± 12.8	33.61 ± 15.67	22.5 ± 3.5	0.006
	PI-LL	12.9 ± 13.21	15.52 ± 12.01	26.23 ± 18.3	44.67 ± 14.9	0.0001

^α^ Calculated with ANOVA test.

**Table 3:** Significant Correlation between Pre-Intervention Parameter and Post-Intervention Change ^α^.

	Post-Intervention Change	VAS	ODI	SVA	PT	LL	PI-LL
Pre-Intervention							
**VAS**		−0.444 *	−0.104	−0.04	−0.151	0.034	−0.038
**ODI**		−0.18	−0.43 *	−0.255	0.048	0.104	−0.104
**SVA**		0.268	0.133	−0.646 *	0.097	0.34 *	−0.298
**PI**		0.151	−0.004	−0.231	0.037	0.060	−0.041
**PT**		0.238	0.197	−0.253	−0.317 *	0.226	−0.226
**LL**		−0.236	−0.264	0.260	0.162	−0.4 *	0.411 *
**PI** **-LL**		0.304 *	0.235	−0.374 *	−0.123	0.369 *	−0.394 *

* r value more than 0.3 or less than −0.3; ^α^ Calculated with Pearson correlation coefficient.
